# 111 Crawling Before We Walk: Transdisciplinary Insights into Improving the Liveability and Walkability of Campus Settings

**DOI:** 10.1093/eurpub/ckae114.131

**Published:** 2024-09-26

**Authors:** Lorraine D’Arcy, Eoin McGillicuddy, Caren Gallagher, Leo McConnell

**Affiliations:** Technological University Dublin, Dublin, Ireland; Technological University Dublin, Dublin, Ireland; Office of the Planning Regulator, Dublin, Ireland; Technological University Dublin, Dublin, Ireland

## Abstract

**Purpose:**

Since the 1960s, development patterns influenced by upstream land use planning policies have favoured a more suburban car-based approach for street and neighbourhood design (McGrath, 1992), subsequently influencing transport system needs and scheme design. In turn, transport policy and design influence population physical activity levels and emissions. As we moved to this more suburban design, our university campuses followed suit and now we have challenges that deter physical activity to and through our university campuses. Furthermore, air quality issues encouraged by a suburban design can enhance an individual’s discomfort in participating in physical activity and may influence their decision to drive rather than walk or cycle, exasperating the air quality (An et al., 2018). Central to this transdisciplinary issue are differences in terminology and unfamiliarity with the needs, objectives and processes of other disciplines which can make communication difficult between professional groups (Fitzsimons D’Arcy, 2013). Therefore, the key to delivering walkable and liveable places lies with consulting transdisciplinary practitioners and policy-makers, encouraging the avoidance of siloed working practices (Rutter et al., 2017).

**Methods:**

Building upon the findings of the World Café at the Walk21 Ireland conference in 2022, the research team seek to understand the challenges and needs within Ireland of a transdisciplinary group of stakeholders from planning, engineering, health and environmental backgrounds. A thematic analysis is applied to collate key themes relating to the challenges and needs faced by stakeholders and how to avoid siloed working practices.

**Results:**

The results will grant the identification of transdisciplinary challenges and needs, laying the foundations of a collective approach to design for and deliver on walkability and liveability which will subsequently be applied using TU Dublin’s campuses as a testbed. Finding opportunities to improve the walkability and liveability of suburban campuses in a more cohesive approach can have transferability of good practice to other suburban campus settings including employment and healthcare, while having a resounding impact on population health and wellbeing through enhancing physical activity.

**Support/Funding Source:**

This project is funded under the Science Foundation Ireland National Challenge Fund Sustainable Communities Challenge

